# Motility and Mechanical Properties of Dendritic Cells Deteriorated by Extracellular Acidosis

**DOI:** 10.1007/s10753-020-01373-z

**Published:** 2020-10-31

**Authors:** Lu Tong, Ping Yue, Yingying Yang, Jin Huang, Zhu Zeng, Wei Qiu

**Affiliations:** School of Biology and Engineering, Guizhou Medical University, Guiyang, 550025 China

**Keywords:** extracellular acidosis, dendritic cells, motility, cellular mechanical properties

## Abstract

Dendritic cells (DCs) are the most powerful antigen-presenting cells known to date and play an important role in initiating and amplifying both innate and adaptive immune responses. Extracellular acidosis is an important hallmark of a variety of inflammatory processes and solid tumors. However, few studies have focused on the effect of extracellular acidosis on DCs and their functions. Cellular mechanical properties reflect the relationship between cell structure and function, including cytoskeleton (especially F-actin organization), membrane negative charges, membrane fluidity, and osmotic fragility*.* The study investigated the effects of extracellular acidosis on the DCs functions from the perspective of cellular migration and mechanical properties. The results showed that migration ability, F-actin contents, and membrane negative charges of DCs were reduced by extracellular acidosis no matter whether LPS stimulated its maturation or not. And these functions could not return to normal after removing acidic microenvironment, which revealed that the function impairment induced by extracellular acidosis might be irreversible. In addition, the proliferation capacity of stimulated allogeneic T cells was impaired by extracellular acidosis. Our results suggest extracellular acidosis may play an immunosuppressive role in DCs-mediated immune process.

## INTRODUCTION

DCs are the most potent antigen-presenting cells that play a crucial role in initiating and amplifying both the innate and adaptive immune responses. Functionally, DCs undergo two stages of differentiation: immature DCs (imDCs) and mature DCs (mDCs). The imDCs reside in non-lymphoid tissues and specifically capture and process foreign antigens. Then, they travel through blood or lymph to secondary lymphoid organs and gradually differentiate to mDCs, physically interacting with naive T cells to initiate immune responses or tolerance [1, 2]. It has been reported that the motility and immune functions of DCs have deteriorated in numerous inflammatory diseases and tumors [3–5].

Extracellular acidosis (or acidic microenvironment) is a significant hallmark of various inflammatory processes and solid tumors. The potential of hydrogen (pH) is an important physiological indicator of homeostasis *in vivo*, which usually ranges from pH 7.2 to 7.4 in the physiological state. However, interstitial acidification is commonly associated with the development of inflammatory reactions against pathogens in peripheral tissues, ranging from pH 5.5 to 7.0 [6–8]. Autoimmune processes such as rheumatoid arthritis and asthma are also associated with lower pH in injured tissues compared with normal tissues [9, 10]. In addition, observations made in solid tumors such as breast cancer, malignant melanomas, squamous cell carcinomas, brain tumors, adenocarcinomas, and sarcomas showed that tumor microenvironments reach pH ranging from 5.8 to 7.4 [6]. Importantly, low pH has been shown to favor cancer progression by promoting local tumor invasion and distant metastatic spread [11]. Nowadays, different strategies have been explored to utilize the relative acidity of tumor *versus* normal tissue in order to enhance the efficacy of antitumor therapy [12, 13].

Although the acidic microenvironment is a common and significant feature in a variety of inflammatory processes and tumors, few studies analyze effect of extracellular acidosis on immune cells, especially DCs. There were only two reports about effects of extracellular acidosis on the functions of murine bone marrow–derived DCs, and the results showed that acidification of the culture medium to pH 6.5 enhanced the endocytosis and expression of cell surface proteins involved in Ag presentation of DCs. This effect appears to be mediated by acid-sensing ion channels, which represent a family of Na^+^ channel and are activated by extracellular protons [14, 15].

Cellular mechanical properties reflect the relationship between cell structure and function, including cytoskeleton organization, membrane negative charges, membrane fluidity, osmotic fragility, and *etc.* The motility and mechanical properties of DCs are closely related to the immunological function such as antigen presentation and stimulation of T cells [16–19]. Hence, the study investigated the effects of extracellular acidosis on the DCs functions from the perspective of cellular motility and mechanical properties. Because imDCs capture and process antigens in peripheral tissues, and considering that many inflammatory processes as well as the growth of multiple tumors lead to the development of the acidic microenvironment in peripheral tissues, we examined the impact of extracellular acidosis on the function of DCs based on the following aspects in the study: (1) effect of extracellular acidosis on imDCs and their maturation process. (2) The functional changes after DCs migrate out of the acidic microenvironment. Our results show that extracellular acidosis reduces the migration, F-actin contents, and membrane negative charges of DCs, but has no effect on its membrane fluidity and osmotic fragility (data not shown). These results help us better understand the immune regulatory function of DCs and provide important information for preventing various inflammations and tumors.

## MATERIALS AND METHODS

### Animals

The 6–8-week-old C57BL/6 mice were purchased from the Animal Experiment Center of Guizhou Medical University. They were housed under specific pathogen-free conditions and kept at 22 ± 2 °C. Animal care and treatment were performed in accordance with the Animal Care Welfare Committee of Guizhou Medical University.

### DCs Cultures and Treatments

The femurs and tibias obtained from euthanized mice were washed twice with phosphate-buffered saline (PBS). Both ends of the bones were cut with sterile scissors, and then, the marrow was flushed out using RPMI 1640 (Gibco, NY, USA) and filtered into a sterile conical tube (Corning, NY, USA). Subsequently, the liquid was centrifuged for 5 min at 1200 rpm, and then, supernatant was removed and resuspended in red blood cell lysis buffer (Solarbio, Beijing, China) 5 min to lyse red blood cells. After washed twice with PBS, the cells were cultured for 7 days in RPMI1640 medium supplemented with 10% fetal bovine serum (Gibco, NY, USA), 20 ng/ml recombinant murine GM-CSF (PeproTech, Rocky Hill, USA), and 10 ng/ml recombinant murine IL-4 (PeproTech, Rocky Hill, USA) to obtain imDCs. The imDCs were induced with 100 ng/ml lipopolysaccharide (LPS; InvivoGen, Toulouse, France) for 24 h to obtain mDCs.

To study the effects of extracellular acidosis on the migration capacity and mechanical properties of imDCs and their maturation processes, the imDCs were divided into four groups added medium with pH 7.3, pH 6.5, pH 7.3 + LPS, and pH 6.5 + LPS. The medium at pH 7.3 was adjusted to pH 6.5 by addition of isotonic HCl. The pH 7.3 and pH 7.3 + LPS groups were used as the control group of pH and pH + LPS group, respectively. The cells from pH 7.3 and pH 6.5 groups were respectively collected at 1 h, 4 h, and 24 h after adding different pH media. The pH 7.3 + LPS and pH 6.5 + LPS group cells were collected only at 24 h after adding different pH and 100 ng/ml LPS media. The collected cells were performed for the experiments on mobility and cellular mechanical properties. To study the functional changes after DCs migrate out of the acidic microenvironment, imDCs were incubated for 4 h at pH 6.5 and then the culture medium was replaced with pH 7.3 or pH 7.3 + LPS. The imDCs were divided into four groups added medium with pH 7.3, pH 6.5 → 7.3, pH 7.3 + LPS, and pH 6.5 → 7.3 + LPS, and the similar method as above was used.

### Cell Viability Assay

The Counting Kit-8 (CCK-8, Solarbio, Beijing, China) was used to quantify the cell viability. The cells were incubated in a 96-well plate (1 × 10^4^/well) for 24 h and then incubated with 10 μl of CCK-8 solution for 2 h at room temperature. The absorbance was measured spectrophotometrically at 450 nm.

### Motility

Transwell system was used to determine the motility of DCs. The cells were placed in the upper Transwell chamber (Corning, NY, USA) with 8.0-μm pores and the lower compartment filled with RPMI 1640 medium. In the LPS-induced group, the medium of lower compartment was supplemented 100 ng/ml CCL19 (BioLegend, San Diego, USA) as chemokine for mDCs. In the pH group, the chemokine was not used. The cells in the lower compartment were collected and the numbers were counted using a cell counting plate. The ratio of the counted numbers to the initial adding amount represents the migration percentage. Three repeated experiments were performed for standard deviation (SD) analyses.

### Confocal Laser Scanning Microscopy Analysis

The cells were immobilized on the coverslips coated with poly-l-lysine (Solarbio, Beijing, China) for 30 min and fixed with 4% paraformaldehyde (Solarbio, Beijing, China). After permeabilization with 0.1% Triton X-100 (Solarbio, Beijing, China), DCs were incubated with PBS containing 1% bovine serum albumin (Solarbio, Beijing, China) for 30 min. Subsequently, the cells were stained with 2 U rhodamine phalloidin (Solarbio, Beijing, China) in the dark for 20 min, followed by staining with 4,6-diamidino-2-phenylindole (Solarbio, Beijing, China) in dark for 5 min. Then, the coverslips were mounted with antifade medium (Solarbio, Beijing, China) on glass slides and the cells were imaged by confocal laser scanning microscopy (FV1000, Olympus, Tokyo, Japan). The 3-dimensional images were reconstructed with the software function of Olympus. The F-actin contents were quantified by measuring the mean fluorescent intensities using the ImageJ software. Three repeated experiments were performed and about six cells from each group were randomly selected for analyses in each experiment.

### Electrophoretic Mobility

The collected cells were adjusted to 2 × 10^6^/mL with 9% (w/v) sucrose solution. The electrophoretic mobility (EPM) was examined by a cell electrophoresis meter (WD-9408E, Liuyi Biotechnology, Beijing, China) at 30 °C. Ten cells were randomly selected for each group and three repeated experiments were performed to obtain the means.

### Mixed Leukocyte Reaction

The mixed leukocyte reaction (MLR) assay was performed to measure the immune stimulatory capabilities of mDCs. Allogeneic T cells were obtained from the murine spleen through the nylon wool columns (Corning, NY, USA). A total of 2 × 10^5^ T cells were cultured with the same number of DCs incubated at 37 °C with 5% CO_2_ for 48 h, and then subjected to CCK-8 analysis as described above.

### Statistical Analysis

The mean and SD from the three repeated experiments were calculated. Student’s *t* test was used to compare the two means using Microsoft Excel. Differences were considered to be significant at *p* < 0.05 and to be highly significant at *p* < 0.01.

## RESULTS

### The pH 6.5 Treatment Did Not Affect DCs Viability

**Fig. 1:**
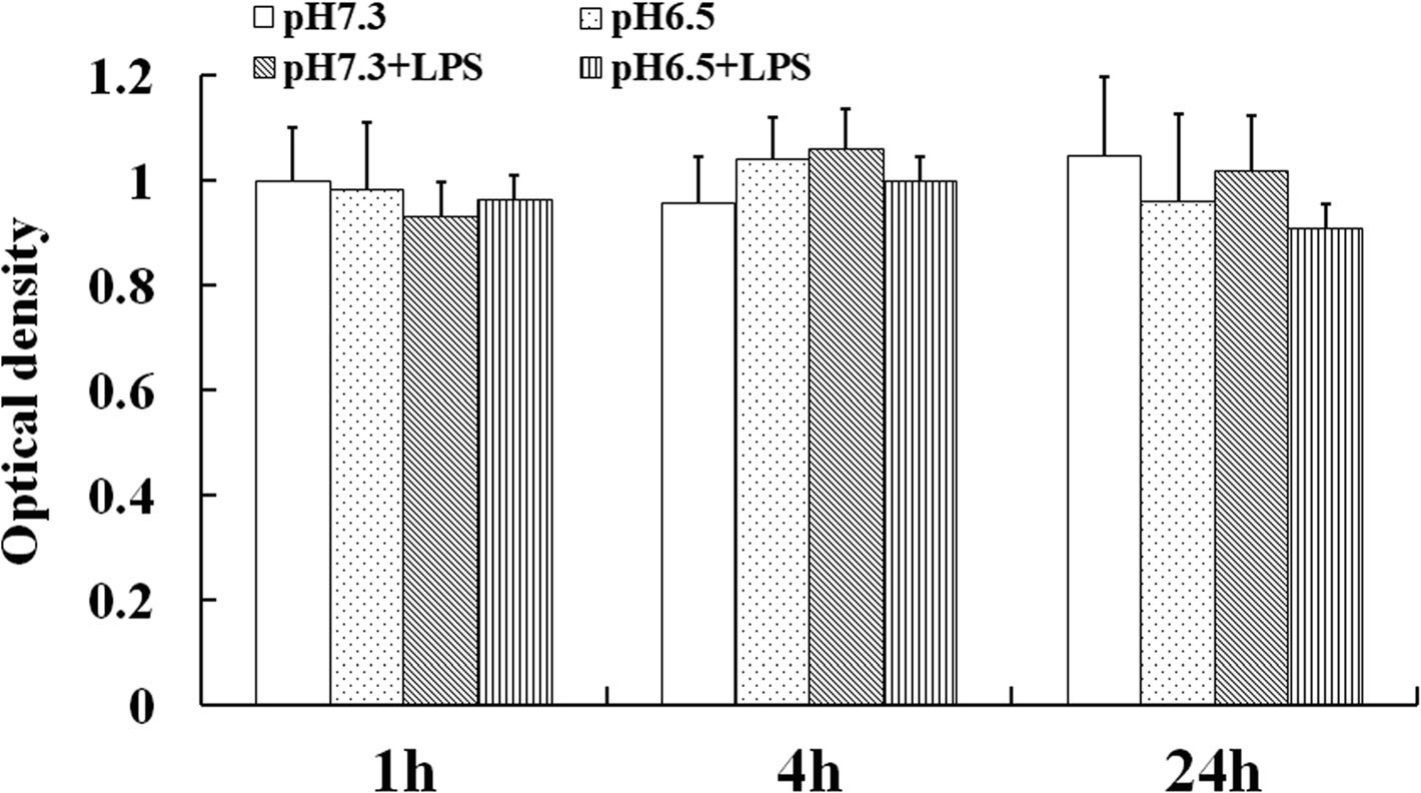
The viability of DCs was not altered in the acidic microenvironment. The viability of the pH 7.3 group at the 1 h was set to 1.0. Each column in the histogram represents the mean value of three samples, and the short line on the histogram represents the standard deviation of the mean value of three samples.

The cell viability is closely related to its function. So far, all of the studies on the regulation of DCs function by extracellular acidosis use pH 6.5 as the treatment condition and have achieved remarkable results [13]. In the study, our results showed that the DCs viability was not affected after treated with pH 6.5 (Fig. 1). Therefore, pH 6.5 was selected to study the effect of extracellular acidosis on DCs function in the experiment.

### Extracellular Acidosis Reduced the Motility

**Fig. 2:**
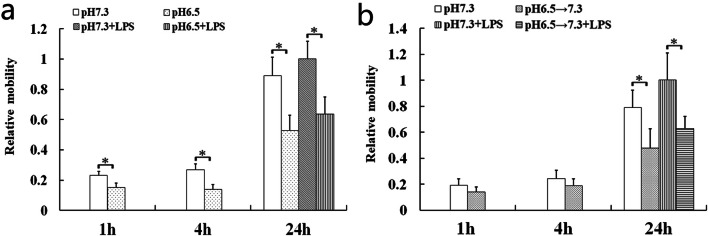
The migration capabilities of DCs were impaired by extracellular acidosis no matter whether LPS stimulated its maturation or not. **a** DCs in the acidic microenvironment. **b** DCs leaving the acidic microenvironment. Migration percentage of the pH 7.3 + LPS group at the 24 h after acidosis treatment (**a**) and removing acidosis (**b**) was set to 1.0. Each bar represents the mean ± SD of the three experiments. Statistically significant differences were represented with asterisks (*p < 0.05).

Excellent motility of DCs is crucial for their performing immune functions, including antigen-uptaking in peripheral tissues and acquiring antigen presentation in lymph nodes [2]. The results showed that the migration capability of DCs was markedly diminished in the acidic microenvironment (Fig. 2a). Tong *et al*. have reported that exposure to pH 6.5 for 4 h resulted in a significant difference of DCs immune function [14]. Therefore, DCs were incubated at pH 7.3 after exposure to pH 6.5 for 4 h to simulate the process of DCs that migrate out of the acidic microenvironment to the lymph node. Our data revealed that the DCs mobility also was impaired after leaving the acidic microenvironment (Fig. 2b).

### Extracellular Acidosis Damaged the F-Actin Structure

**Fig. 3:**
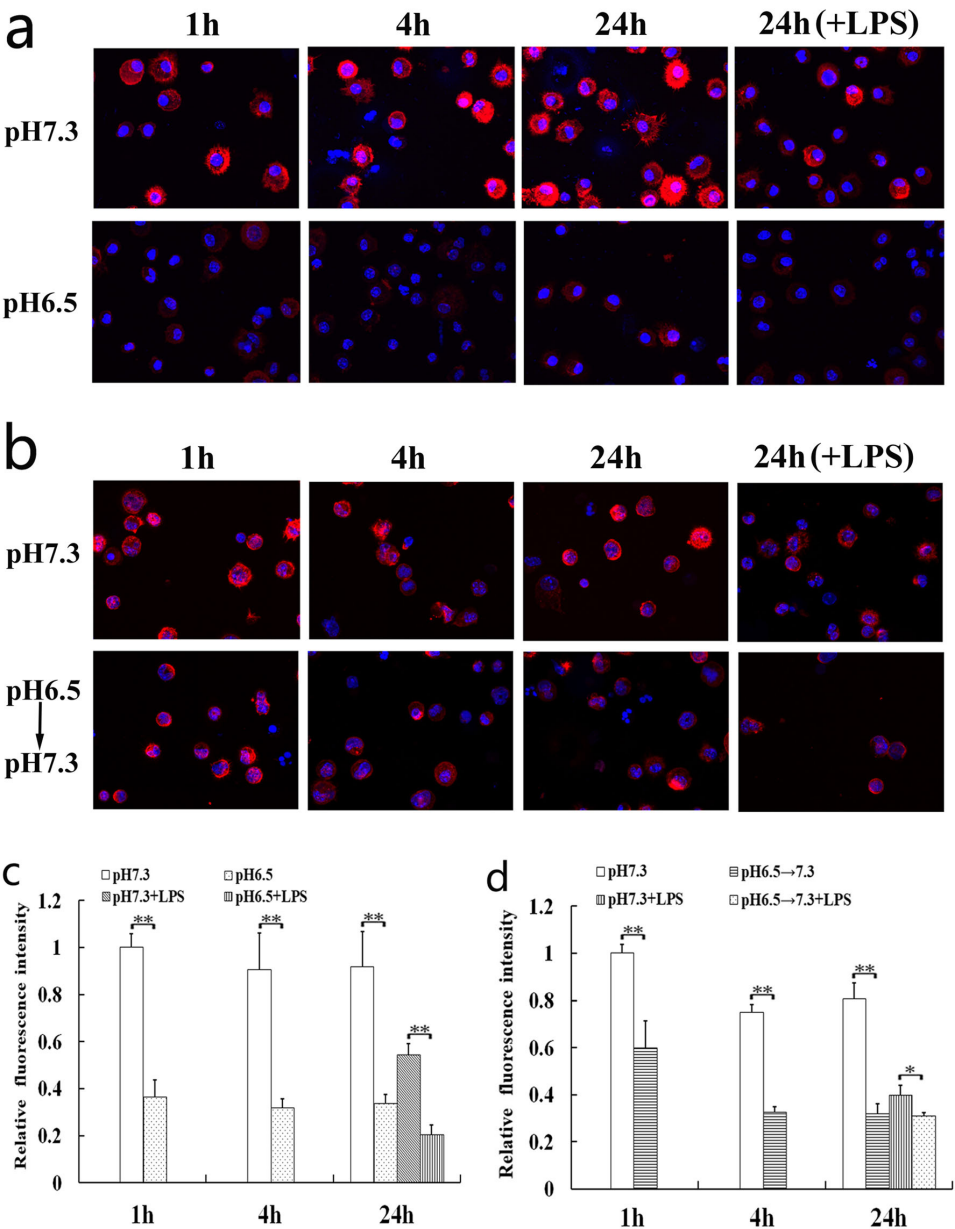
Effects of extracellular acidosis on F-actin organization of DCs. Confocal microscopy analysis was performed on DCs to analyze the effects of extracellular acidosis on F-actin organization in the acidic microenvironment (**a**, **c**) or after removing it (**b**, **d**). **a**, **b** The 3-dimensional images from a representative experiment are shown. The magnification used is × 600. **c**, **d** Results are expressed as mean fluorescence intensity values and represent the arithmetic mean ± SD of three experiments. The value of the pH 7.3 group at the 1 h after acidosis treatment (**c**) and removing acidosis (**d**) was set to 1.0. Statistically significant difference was indicated by bars with asterisks (*p < 0.05; **p < 0.01).

The cytoskeleton of eukaryotic cells is a network structure system composed of protein fibers, mainly including microfilaments, microtubules, and intermediate fibers. Microfilaments composed of F-actin mainly control cell plasticity and migration by generated depolymerization and polymerization [20, 21]. Our result showed that the F-actin contents were significantly reduced by extracellular acidosis (Fig. 3).

### Effects of Extracellular Acidosis on EPM

**Fig. 4:**
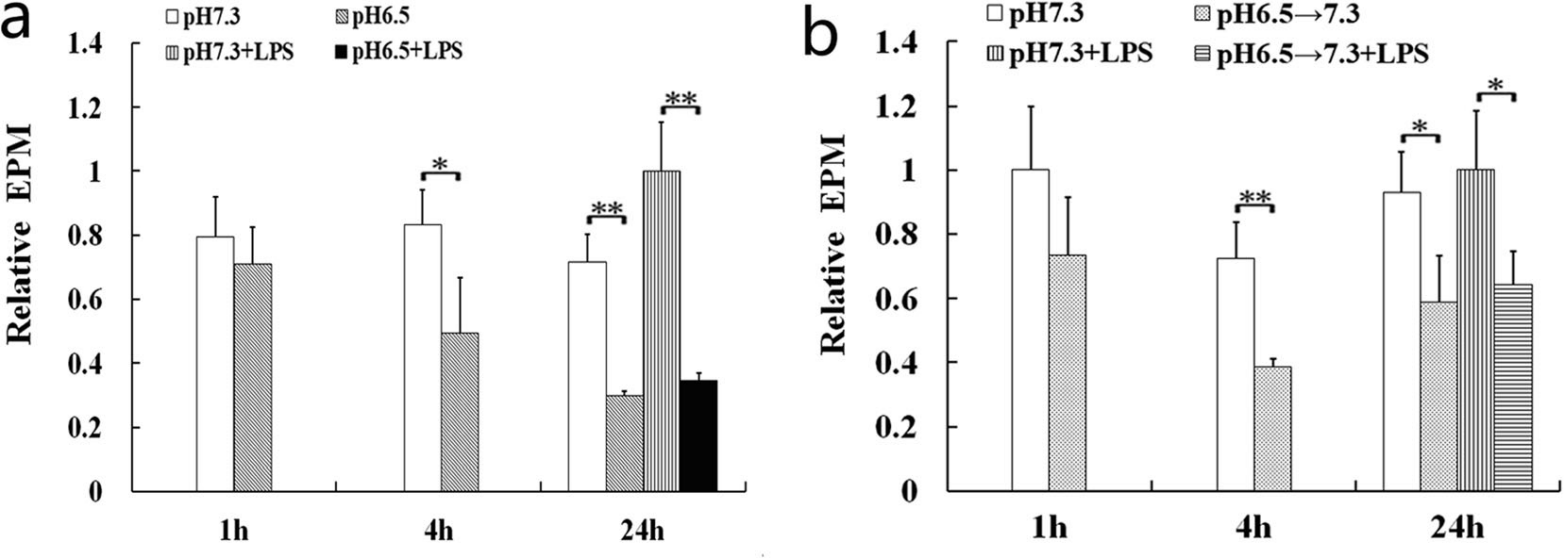
The amount of negative charges of DCs was decreased in the acidic microenvironment (**a**) or after removing the acidic microenvironment (**b**). EPM of the pH 7.3 + LPS group at the 24 h after acidosis treatment (**a**) and removing acidosis (**b**) was set to 1.0. The bars indicated the mean ± SD of EPM. The statistical significance was calculated using Student’s *t* test (*p < 0.05; **p < 0.01).

The EPM indicates that the negative charges come from the hydrolysis of sialic acid of glycoprotein located on the surface of cell membrane [19]. The results showed that EPM of DCs was apparently reduced by extracellular acidosis (Fig. 4). That is, the amount of negative charges of DCs was decreased by extracellular acidosis.

### Extracellular Acidosis Impaired the Ability of Stimulated T Cell Proliferation

**Fig. 5:**
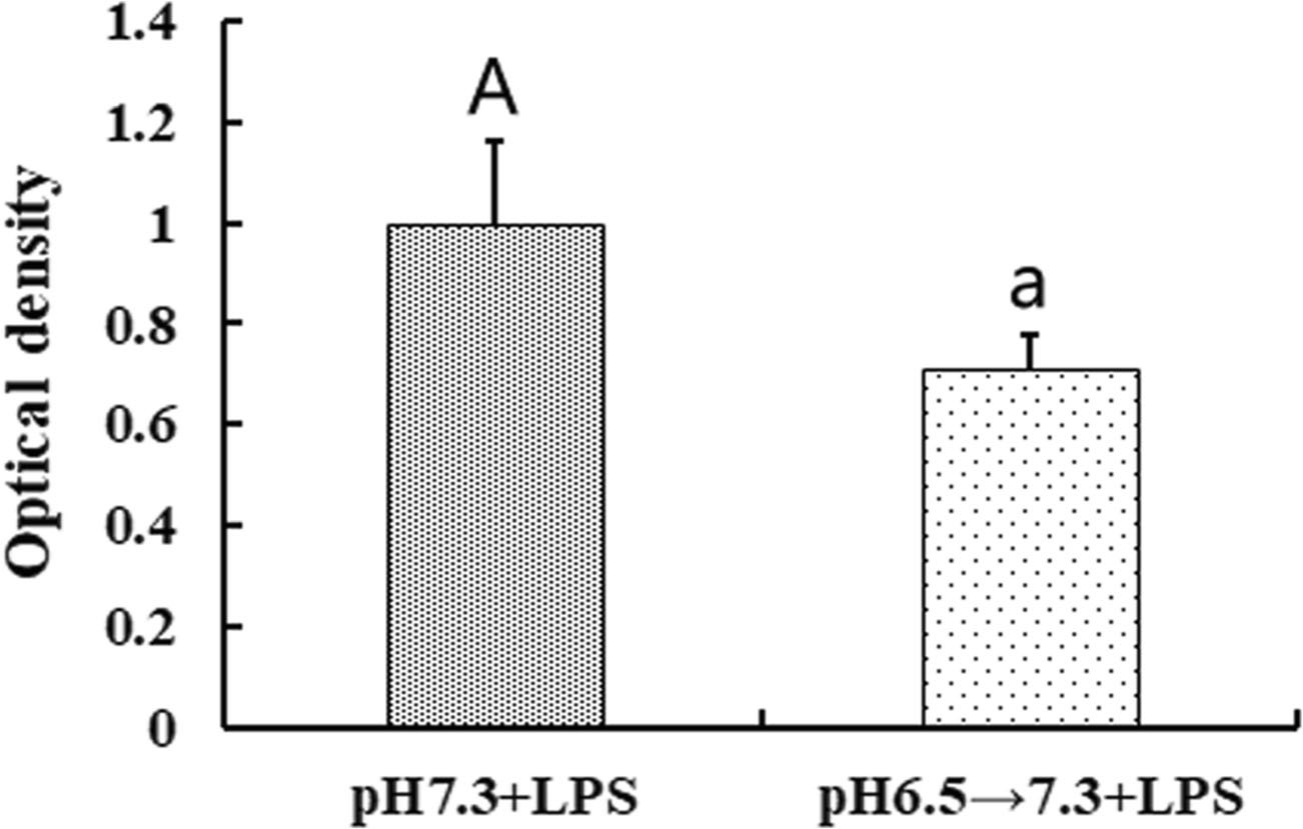
Effect of extracellular acidosis on the immune stimulatory capability of DCs. The result was expressed as proliferating index and the value of the pH 7.3 + LPS group was set to 1.0. Each bar represents the mean ± SD from three independent experiments. Statistically significant differences were indicated by different letters (A, a) (*p* < 0.05).

The main function of mDCs is their stimulatory capabilities, which is determined by the primary allogeneic mixed leukocyte reaction (MLR) assay using murine spleen T lymphocytes as responder cells. The imDCs were induced maturation by LPS incubated in pH 7.3 for 24 h after exposed to pH 6.5 for 4 h. The imDCs were incubated at pH 7.3 for 4 h, followed by LPS-induced maturation for 24 h as controls. The results showed that pre-treatment with pH 6.5 significantly impaired the immune stimulatory capabilities of mDCs (Fig. 5), suggesting that the stimulatory function may be impaired after DCs leave the acidic microenvironment to lymph nodes.

## DISCUSSION

The study investigated the effect of extracellular acidosis on immune function of DCs from the perspective of cellular motility and mechanical properties. Our data show that extracellular acidosis reduces the migration, F-actin contents, and membrane negative charges of DCs, and these functions cannot return to normal after removing the acidic microenvironment, which reveal that the function impairment induced by extracellular acidosis may be irreversible.

DCs migrate to lymph nodes and activate T cells after acquire antigens to induce immune response. In tumor-bearing hosts, the immune function of the body is impaired. A key reason is the downregulation of DCs motility, which makes DCs fail to migrate to the lymph nodes [22]. The development of acidic microenvironment is a prominent characteristic of multiple solid tumors, which has been proven to play a key role in tumorigenesis and progression [11, 23]. Therefore, it is great significance to know the effect of extracellular acidosis on DCs mobility. In our study, DCs migration was impaired by extracellular acidosis, which may be one of the important reasons why DCs fail to migrate to lymph nodes and immune function was impaired in tumor-bearing hosts.

Dynamic organization of actin cytoskeleton is essential to function of DCs, including antigen capture, deformability, and motility, especially F-actin organization [24, 25]. F-Actin is a filamentous polymer composed of multiple globular actin (G-actin) subunits. The dynamics of the intracellular ratio between F-actin and G-actin are vital for actin remodeling [26, 27]. In our experiment, rhodamine phalloidin was used to specifically labeled F-actin, instead of combining it with G-actin. Our results showed that the F-actin contents of DCs were significantly reduced by extracellular acidosis. Interestingly, it was found that the F-actin content significantly decreased within 1 h, indicating that F-actin may be depolymerized once DCs entered the acidic microenvironment. The data also indicated that the re-polymerized of F-actin was suppressed even if DCs left the acidic microenvironment. F-Actin organization of DCs is closely related to its migration ability. These results suggested that extracellular acidosis was likely to affect DCs migration ability by regulating F-actin polymerization. Further investigations about the relationships among acidic microenvironment, DCs migration, and F-actin organization need to be done.

The electrical properties of cell membrane play an important role in cell-cell and extracellular matrix interactions. The cells of eukaryotic organism have negatively charged surfaces *in vivo*, which is related to their adhesion to neighboring cells or to solid organic or inorganic surfaces [19]. DCs and T cells must overcome the barrier posed by negatively charged glycocalyx components for direct physical contact during the process of antigen presentation [28]. Here, the results showed that the surface negative charge of DCs was significantly reduced by extracellular acidosis. Fewer negative charges on the surface of DCs could increase the adhesion between DCs and T cells, which might lead to an increased contact time. The different contact times between DCs and T cells induce different immune responses and the longer contact time prime immune tolerance [17, 18]. Thus, the interaction of DCs with T cells might be disturbed by extracellular acidosis through the decrement of negative charges on DC surfaces, which may be responsible for the immune dysfunction mediated by DCs in many inflammatory diseases and tumors.

The stimulatory capability of mDCs is their most important function for immune efficacy. The short-term exposure of imDCs to acid conditions followed by longer periods at a neutral pH and gradual differentiation to mDCs probably reflects what occurs *in vivo* because the migration of DCs from the periphery to lymph nodes usually demands several hours and occurs at a neutral pH environment [29]. Hence, the maturation of imDCs was induced by LPS incubated in pH 7.3 for 24 h after exposed to pH 6.5 for 4 h and then MLR was performed in our study to simulate the process of DCs *in vivo*. The results showed that the immune stimulatory capabilities of DCs were impaired by extracellular acidosis. In conclusion, extracellular acidosis may play an immunosuppressive role in DCs-mediated immune process from the perspective of cellular motility and mechanical properties.
